# Phylogenetic Characteristics and Low-Temperature Response Expression Patterns of *PmKIN* Gene Family in *Prunus mume*

**DOI:** 10.3390/genes17091151

**Published:** 2026-09-20

**Authors:** Aiqin Ding, Ziwen Geng, Lulu Li, Lu Feng, Peng Wang

**Affiliations:** College of Forestry Engineering, Shandong Agriculture and Engineering University, Jinan 250100, China

**Keywords:** *P. mume*, kinesin, genome-wide identification, low-temperature stress, gene expression

## Abstract

**Background**: Kinesins are ATP-dependent molecular motors that mediate intracellular transport, cytoskeleton remodeling, and abiotic stress responses in plants. The *KIN* gene family remains poorly characterized in *Prunus mume*. This study aimed to explore the evolutionary features and cold-responsive functions of the *PmKIN* gene family. **Methods**: A systematic genome‑wide analysis was performed in *P. mume*. We performed phylogenetic classification, conserved domain detection, gene duplication and selection pressure analysis, cis-element prediction, tissue expression profiling, cold stress expression assay, and protein interaction network prediction. **Results**: Fifty *PmKIN* family members were defined and grouped into 10 subfamilies. K14 subfamily contained the largest number of members. All members harbor conserved motor domains, and segmental duplication drove the expansion of this gene family, which was overall constrained by purifying selection. The *PmKIN* homologous genes were highly conserved among Rosaceae species, including *Prunus persica*, *Prunus armeniaca*, *Prunus avium*, and *Malus domestica*. Our promoter analysis detected abundant cis-elements for light, hormone, cold, and drought signals in *PmKIN* genes, especially in the K14 and K7 subfamilies. These genes displayed tissue-biased expression, with roots and stems showing much higher transcript levels than fruits. Under low-temperature stress, the tolerant cultivar appeared to mount a relatively rapid *PmKIN* upregulation, whereas the sensitive one seemed to show a sluggish response and poor recovery. Three members (*PmKIN22/27/35*) were suggested to be potentially critical in low-temperature-response regulation. A 285-pair interaction network predicted PmKIN45 as the central hub, and functional predictions imply that PmKIN proteins may be linked to microtubules, hormone pathways, and stress signaling, possibly coordinating growth and low-temperature responses. **Conclusions**: This work screens candidate genes associated with low-temperature responses in *P. mume*, providing genetic resources for the molecular breeding of cold-resistant cultivars and expanded cultivation of *P. mume* and other ornamental horticultural species.

## 1. Introduction

Low temperature represents the predominant abiotic stress limiting the biogeographic distribution, annual growth, and establishment of ornamental attributes in woody ornamental plants. Cold and freezing stresses trigger a series of physiological damages in woody plants, including reduced cell membrane fluidity, disturbed intracellular ion balance, and massive accumulation of reactive oxygen species. These stress-triggered physiological disorders further lead to cytoskeletal abnormalities and the suppression of intracellular substance trafficking. Consequently, frost injury to buds, branch dieback, and flowering defects emerge, which significantly compromise plant survival capacity and ornamental landscape utility [1,2].

As a characteristic and traditional ornamental flower of China, *P. mume* has significant ornamental, edible, and cultural value. Its unique trait of blooming in early spring makes it an indispensable plant in landscape architecture [3]. The natural germplasm of *P. mume* is predominantly concentrated in the warm zones of the Yangtze River Basin and southern China. Most cultivars exhibit weak cold adaptability and frequently suffer from winter freezing stress and early spring cold spells under northern open-field cultivation conditions. The northern expansion of *P. mume* cultivation and germplasm utilization has been severely constrained by insufficient cold tolerance [4]. Cold-tolerance research in *P. mume* has examined gene families tied to transcription, Ca^2+^ signaling, and hormone pathways. The ICE-CBF, MAPK, and ABA circuits have been confirmed as central to cold adaptation, yielding numerous candidate genes [5,6]. It is worth noting that previous investigations have predominantly focused on transcriptional regulators. Comparatively, motor-protein families associated with cytoskeleton-dependent intracellular transport are largely overlooked in cold-stress studies of *P. mume*, implying potential uncharted territory within this research domain.

The plant kinesin family not only orchestrates plant growth and developmental processes [7,8,9] but also governs adaptive responses to diverse abiotic stresses such as cold, drought, and salinity [10,11]. Accumulating evidence has confirmed the crucial roles of kinesin family genes in plant cold-stress responses across different species. Under cold stress, *AtKP1* positively modulated seed germination capacity at low temperatures via regulating mitochondrial respiratory activity, thereby constructing a direct regulatory association between kinesin function and cold-responsive energy homeostasis [12]. *OsDLK* functions as a cold signal sensor in *Oryza sativa*, which rapidly translocates from cortical microtubules to the nucleus upon cold stress. It facilitated cold signal cascade transduction and governed the expression of *CBF* pathway-downstream cold-responsive genes [13]. Heterologous expression of the *Allium sativum AsKIN* gene in *Arabidopsis* markedly improved plant antioxidant performance under low-temperature conditions and mitigated freezing-induced cell membrane impairment [14]. Genome-wide identification of the *KIN* family in *Gossypium hirsutum* revealed that the promoters of multiple *GhKIN* members were enriched in low-temperature-responsive cis-elements. And their expression levels were significantly upregulated under low-temperature treatment [15]. Low-temperature stress impairs the plant microtubule cytoskeleton structure, obstructing the intracellular transport of ion transporters, antioxidant-related vesicles, and cold signaling molecules. Kinesins could coordinate cold tolerance by remodeling microtubules and trafficking stress proteins, acting as hubs that link sensing to adaptive responses while maintaining cellular homeostasis [16]. Nevertheless, despite extensive functional evidence from model and crop plants, systematic identification and cold-response characterization of the *PmKIN* gene family remain completely unexplored, representing an important unresolved gap of cold-resistance research in *P. mume*.

Here, we report the first genome-wide investigation of the kinesin gene family in *P. mume*. Beyond routine gene-family annotation, we further assessed cold-responsive expression of *PmKIN* using cold-tolerant and cold-susceptible genotypes together with their hybrid progeny, which provides a unique perspective to evaluate genotype-dependent responses of kinesin genes under low-temperature stress.

In this study, we comprehensively characterized the *PmKIN* gene family through phylogenetic reconstruction, structural and conserved motif analysis, chromosomal mapping and duplication pattern dissection, and promoter cis-element prediction. Transcriptomic datasets derived from roots, stems, leaves, and flower buds were integrated to elucidate the tissue-specific expression characteristics of *PmKIN* genes. Furthermore, qRT-PCR expression profiling was performed on samples from cold-tolerant, cold-susceptible genotypes and their hybrid progeny. These samples were subjected to cold and freezing temperature gradient treatments to screen candidate *PmKIN* genes with significantly low-temperature-induced expression. This study fully reveals the genomic characteristics and cold-responsive expression patterns of the *PmKIN* gene family. It provides candidate genes and a theoretical basis for further dissecting the molecular mechanisms underlying kinesin-mediated low-temperature responses and cold tolerance in *P. mume*. Additionally, this study can supply valuable genetic resources and innovative research perspectives for the molecular breeding of cold tolerance and the development of new cold-resistant *P. mume* cultivars as well as other ornamental horticultural plants.

## 2. Materials and Methods

### 2.1. Identification of PmKIN Gene Family

Genomic and proteomic data of *P. mume* were downloaded from the NCBI database (https://ncbi.nlm.nih.gov/?hl=ru) (accessed on 5 March 2026) [17]. Genome sequences of *P. persica*, *P. armeniaca*, *P. avium*, and *M. domestica* were obtained from the GDR database (https://www.rosaceae.org/) (accessed on 5 March 2026), while those of *Arabidopsis thaliana* were obtained from TAIR (https://www.arabidopsis.org/) (accessed on 7 March 2026). The hidden Markov model (HMM) for kinesin domain PF00225 was further collected from the InterPro database (https://www.ebi.ac.uk/interpro/) (accessed on 9 March 2026) [18]. HMM search was performed using TBtools software v2.466, with an E-value threshold of ≤10^−5^ to preliminarily identify protein sequences of the PmKIN. The SMART web server (https://smart.embl.de/) (accessed on 9 March 2026) was applied to check domain completeness, and only KIN genes carrying full-length functional domains were selected for subsequent experiments [19].

Using ExPASy ProtParam (https://web.expasy.org/protparam/) (accessed on 10 March 2026) and WoLF PSORT, we analyzed the physicochemical properties (length, MW, pI, hydropathicity, instability index, and GRAVY) and subcellular localization, respectively, of all PmKIN proteins [19].

### 2.2. Phylogenetic Relationships, Gene Structure, and Conserved Motif Analysis

Phylogenetic analysis combining kinesin protein sequences of *P. mume*, *P. persica*, *P. armeniaca*, and *A. thaliana* was conducted to resolve the classification patterns of the *PmKIN* family. The Muscle program within MEGA 7.0 was used for multiple sequence alignment, and an ML phylogenetic tree was built with 1000 bootstrap replicates to evaluate tree robustness [20].

Gene structural characterization of *PmKIN* was achieved through the Visualize Gene Structure (from GTF/GFF3 file) function of TBtools. To dissect conserved protein motifs, the Simple MEME Wrapper toolkit was employed, and the maximum number of output motifs was predefined as 10. The conserved protein motifs were further graphically exhibited by the Visualize Motif Pattern module [21].

### 2.3. Chromosomal Localization and Collinearity Analysis

The Advanced Circos module within TBtools was applied for chromosomal localization analysis, and the chromosomal distribution map of target genes was generated for visualization [21]. Genome-wide intraspecific syntenic collinearity analysis for *P. mume* was implemented based on the MCScanX algorithm. Interspecific collinearity analysis among *P. mume*, *A. thaliana*, *P. persica*, *P. armeniaca*, *P. avium*, and *M. domestica* was also conducted. Subsequent graphical visualization of the syntenic relationships was achieved using the embedded Dual Systeny Plot module of TBtools. Meanwhile, Ka, Ks, and Ka/Ks were obtained via the Simple Ka/Ks Calculator of TBtools software v2.466 (NG algorithm), with the latter used to infer selection pressure. Duplication times (T) were estimated from Ks using T = Ks/(2λ × 10^6^) Mya (λ = 1.5 × 10^−8^ for dicots) [22,23].

### 2.4. Cis-Element Identification of PmKIN Promoters and Protein Interaction Analysis

Promoter cis-regulatory motif profiling for *PmKIN* genes was executed through Plant CARE (https://bioinformatics.psb.ugent.be/webtools/plantcare/html/) (accessed on 15 March 2026) [24]. After consolidation of all retrieved analytical data, the Simple Bio sequence Viewer function of TBtools was adopted to generate schematic visual profiles exhibiting the positional distribution of cis-acting elements across *PmKIN* gene promoters. The protein–protein interaction network of kinesin proteins was predicted and analyzed via the STRING database (https://cn.string-db.org) (accessed on 10 May 2026). Subsequently, the retrieved PPI datasets were imported into Cytoscape v.3.9.1 for layout optimization and esthetic refinement [25].

### 2.5. PmKIN Genes Expression Analysis

To examine the expression behavior of *PmKIN* genes, we obtained raw RNA-seq data from the NCBI SRA database (SRP014885), which included samples from buds, fruits, leaves, stems, and roots. Based on FPKM values, heatmaps were generated using TBtools v2.466 [26] and iTOL (https://itol.embl.de/) (accessed on 25 May 2026) [27] to illustrate the tissue distribution and the transcriptional changes triggered by low-temperature stress.

### 2.6. Plant Materials and Low-Temperature Stress Treatments

To characterize the cold-responsive regulatory network of *PmKIN* genes, one-year-old grafted seedlings of ‘Danfenghou’ (apricot mei, the cold-resistant female parent, ‘DFH’), ‘Beijing Yudie’ (true mei, cold-susceptible male parent, ‘BJYD’), and ‘Xiangruibai’ (their hybrid progeny, ‘XRB’) were subjected to low-temperature stress assays [28,29]. All seedlings were grafted onto *Prunus sibirica* rootstock. For each genotype, three independent grafted seedlings were used as biological replicates. All plant materials were cultured under standardized and stable growth parameters: 60–70% soil moisture, 65–70% relative air humidity, a 14 h light/10 h dark cycle, and a light intensity of 150 μmol∙m^−2^∙s^−1^.

Then the stems were subjected to seven low-temperature treatments with a cooling rate of 1 °C/h: (1) control maintained at 25 °C; (2) continuous cold treatment at 4 °C for 8 h; (3) continuous cold treatment at 4 °C for 5 d; (4) short-term freezing exposure at −5 °C for 1 h; (5) recovery at 4 °C following the −5 °C freezing stress; (6) non-acclimated stems exposed to −5 °C for 1 h; and (7) 4 °C recovery for the non-acclimated stems after −5 °C freezing exposure. In this study, we extracted the expression matrix from a published dataset [29] to characterize the expression profiles of *PmKIN* family members.

To dissect the dynamic regulatory profiles of *PmKIN* genes during cold-stress stimulation, we conducted fine time-series cold and freezing treatments on *P. mume* stems. For time-series cold treatment, annual branches of ‘BJYD’ were sampled in September and cultivated in a 4 °C thermostatic incubator. Bud-excised stem segments were harvested at serial time points of 0, 2, 4, 8, 16, 24, 48, and 72 h. For the gradient freezing treatment, branch tissues were collected in November at an ambient minimum temperature of 5 °C. After overnight pre-cooling at 4 °C, the samples were processed with a stepwise cooling regime (1 °C/h) ranging from 0 °C to −5 °C, and stem samples were collected at 0, 2, 4, 6, 8, 10, 24, and 48 h under constant −5 °C conditions. All fresh samples were snap-stored at −80 °C for downstream RNA extraction.

### 2.7. RNA Isolation and Quantitative Real-Time PCR Assays

For quantitative expression analysis, total RNA was isolated from cold-stressed stems with the Takara RNA Extraction Kit (Beijing, China). After DNase treatment to remove genomic DNA, 1 μg of RNA was converted into cDNA using the PrimeScript™ RT Reagent Kit with gDNA Eraser (Takara, Dalian, China). qPCR amplifications were conducted in 20 μL volumes containing cDNA (2 μL) and TB Green II Premix Ex Taq (Takara, Dalian, China). For relative quantification, we used the 2^–ΔΔCt^ method with *PmPP2A* as the internal reference [30]. Each qRT-PCR reaction was performed in triplicate technical replicates. Statistical significance was assessed using Duncan’s multiple range test in SPSS 24.0, and the significance threshold was set to *p* < 0.05. Primer sequences for the three target genes are provided in Supplementary Table S1.

## 3. Results

### 3.1. Identification and Physicochemical Property Analysis of the PmKIN Gene Family

We predicted 50 kinesin genes in the *P. mume* genome, which were sequentially named *PmKIN1* to *PmKIN50* based on their chromosomal positions (Supplementary Table S2). The physicochemical properties of the PmKIN proteins showed substantial variation. Proteins lengths ranged from 362 aa (PmKIN33) to 2241 aa (PmKIN23), with corresponding MWs from 40.71 to 255.09 kDa. Values of pI varied across 5.05–9.67, where acidic proteins accounted for the majority (36 of 50, pI < 7.0). Subcellular localization prediction showed that 33, 11, 5, and 1 PmKINs existed in the nucleus, chloroplast, cytoplasm, and plasma membrane, respectively. This varied organellar distribution implies functional specialization among *PmKINs*.

### 3.2. Phylogenetic Analysis of the PmKIN Gene Family

**Figure 1:**
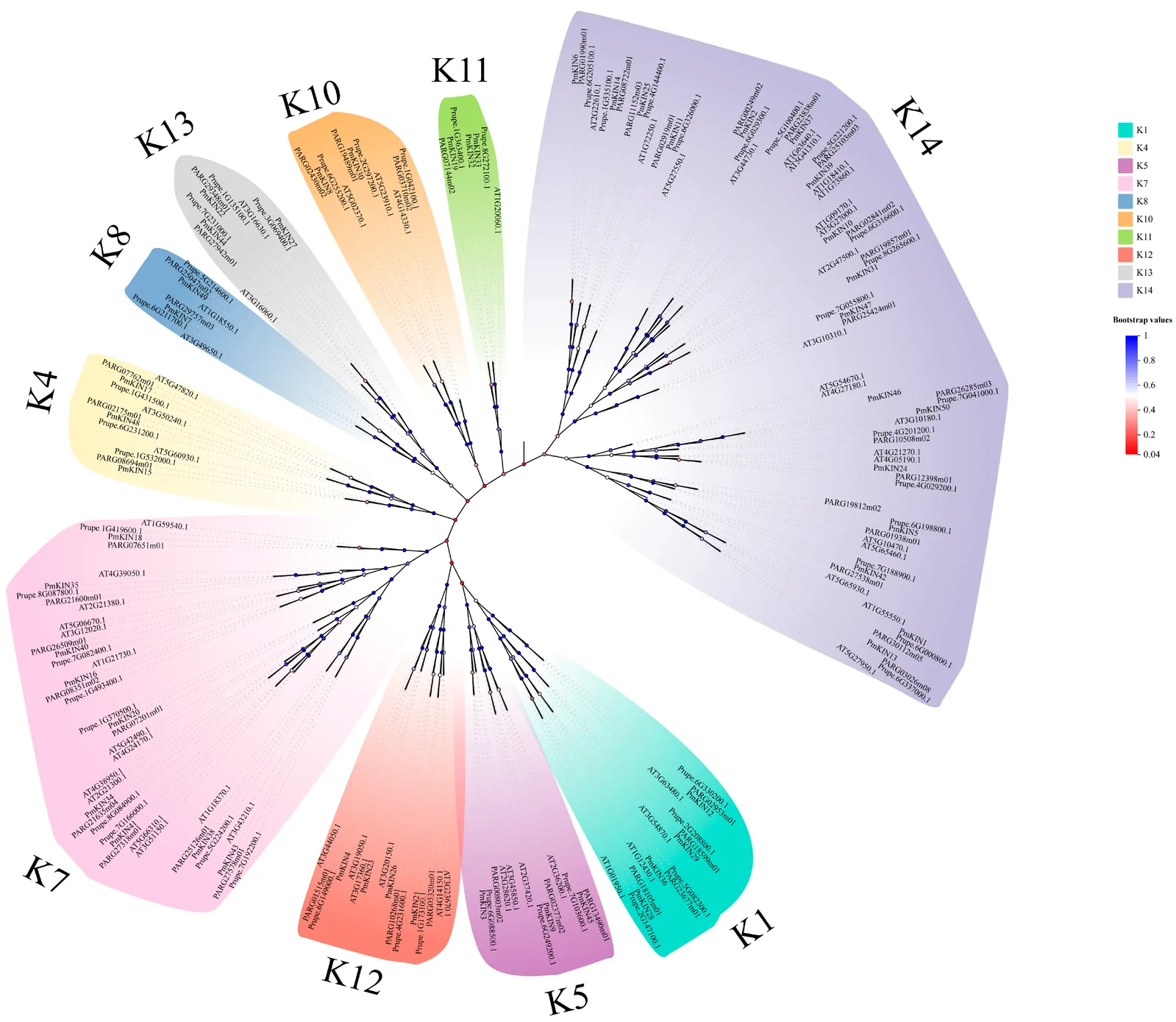
Phylogenetic analysis of the kinesin gene family from *P. mume*.

The evolutionary relationships among members of the *PmKIN* gene family were further investigated by aligning the protein sequences of 208 kinesin genes derived from *P. mume*, *A. thaliana*, *P. persica*, and *P. armeniaca*, and a corresponding phylogenetic tree was subsequently constructed. Based on the classification of *A. thaliana*, we performed phylogenetic clustering of *PmKIN* (Figure 1). The *PmKIN* gene family was classified into 10 subfamilies, including K1 (4 genes), K4 (3 genes), K5 (3 genes), K7 (9 genes), K8 (2 genes), K10 (2 genes), K11 (3 genes), K12 (4 genes), K13 (3 genes), and K14 (17 genes). K14 represented the largest subfamily, while the K8 and K10 subfamilies had the fewest members. Phylogenetic analysis revealed a high sequence similarity of kinesin proteins between *P. mume, P. persica*, and *P. armeniaca,* as well as *A. thaliana*. These proteins clustered into orthologous clades, implying that the corresponding genes retain analogous biological functions.

Clades labeled by Roman numerals denoted different subfamilies of *PmKIN* members. The prefixes AT, Pm, Pr, and PA referred to *A. thaliana*, *P. mume*, *P. persica*, and *P. armeniaca*.

### 3.3. Structural Organization and Conserved Motif Analysis of the PmKIN Gene Family

**Figure 2:**
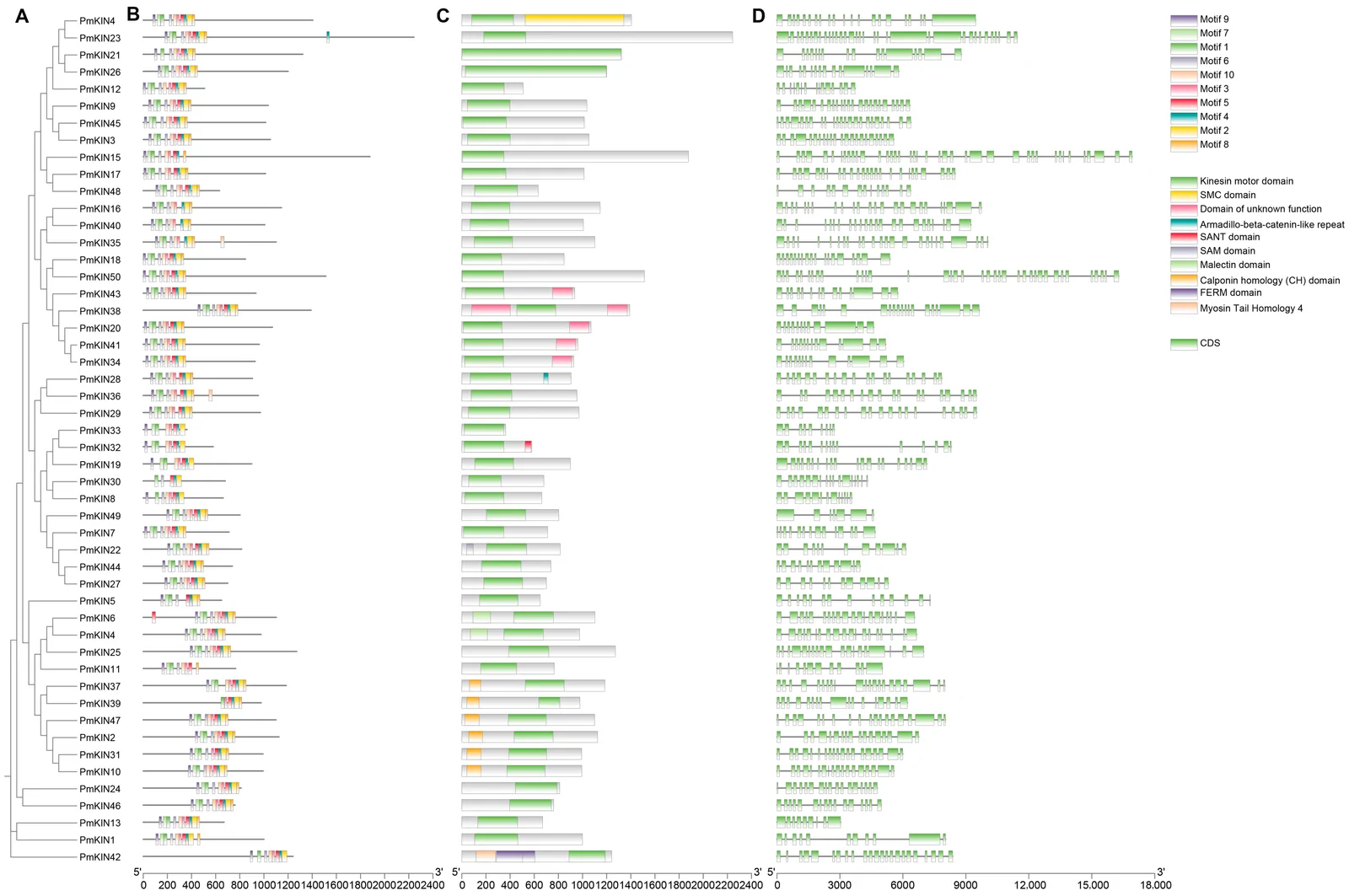
Phylogenetic relationships, conserved motifs, functional domains, and gene structural analysis of *PmKIN* family. (**A**) Phylogenetic tree of PmKIN proteins. (**B**) Distribution of conserved motifs within PmKIN proteins. Ten distinct conserved motifs are indicated by numbered colored boxes (1–10). (**C**) Domain architecture of PmKIN proteins. Core kinesin motor domain is marked with green rectangles, and additional colored rectangles denote auxiliary fused domains. (**D**) Schematic diagram illustrating exon–intron organization of *PmKIN* genes. Introns are shown as gray lines, and exons are depicted as green boxes.

We analyzed the conserved motifs, domain architectures, and gene structures of the 50 *PmKIN* members (Figure 2). Ten conserved motifs (Motif 1–10) were identified, all showing high sequence conservation. Motifs 1 and 7 were present in all PmKINs (Figure 2A,B), where several other motifs showed clade-specific distribution, suggesting functional divergence across distinct subfamilies. All PmKINs contained the typical kinesin motor domain. Moreover, several members carried accessory domains, including Armadillo/β-catenin-like repeats, MyTH4, SAM, SMC, SANT, and CH domains. Their presence and combinations varied across phylogenetic clades. Motif compositions, exon–intron organizations, and domain architectures were largely conserved with a single phylogenetic branch (Figure 2B–D). By contrast, the differences in the structural organization of phylogenetic branches drove functional divergence throughout the *PmKIN* gene family.

### 3.4. Chromosomal Localization and Gene Duplication of the PmKIN Gene Family

**Figure 3:**
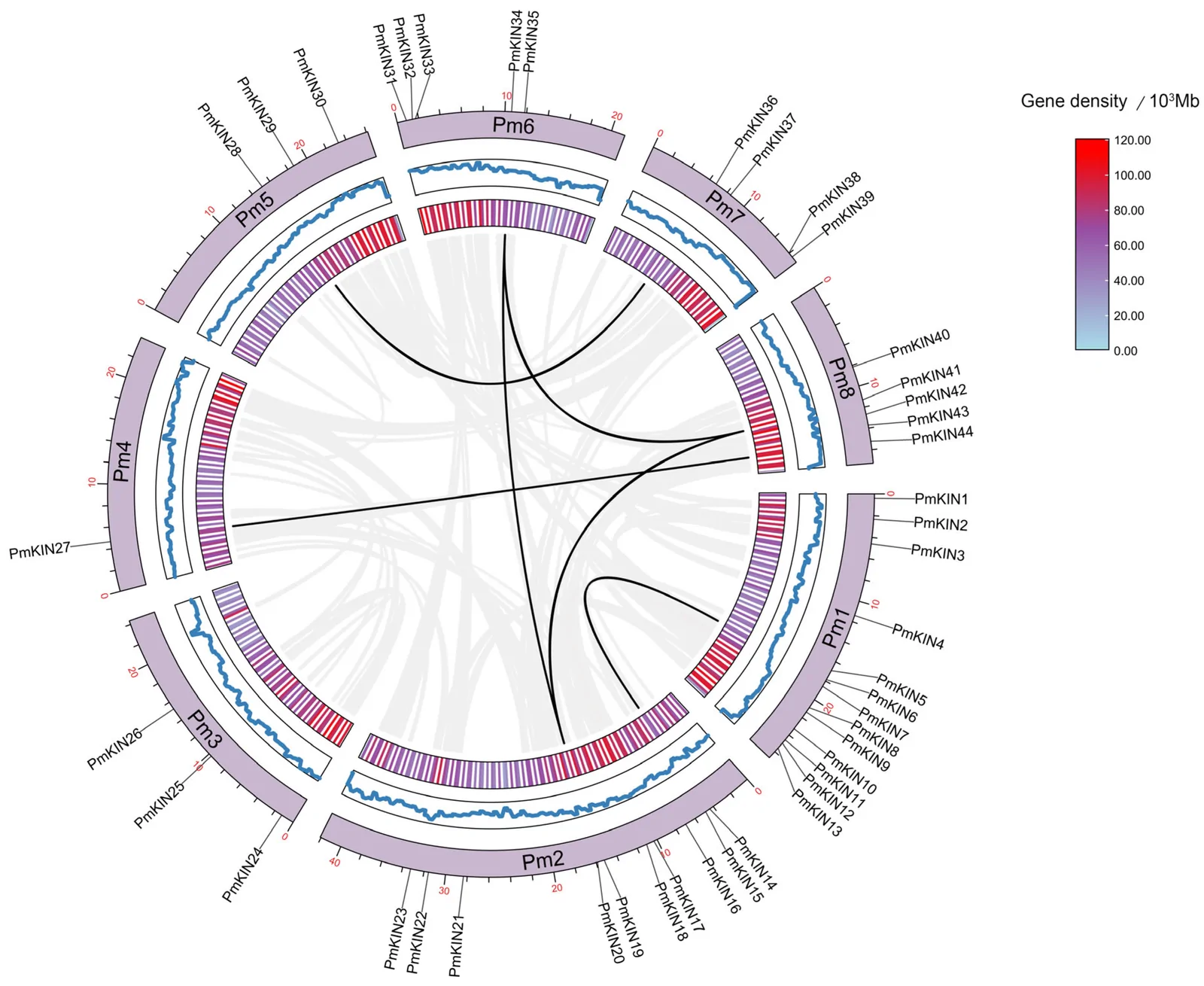
Chromosomal localization and intraspecific collinearity analysis of *PmKIN* genes.

The genomic distribution of *PmKIN* genes was investigated according to chromosome localization (Figure 3). We found that 44 *PmKINs* were localized to eight chromosomes (Pm1–Pm8), while 6 *PmKINs* lacked definite chromosomal positions (Supplementary Figure S1). The 44 mapped *PmKINs* showed irregular clustering on eight chromosomes, with significant differences in gene density between chromosomes. Pm1 carried the highest number of *PmKIN* genes (13 in total), which is in stark contrast to the Pm4 chromosome, which only had one family member. In addition, no tandem duplication events of *PmKIN* genes were observed, implying that the contribution of tandem duplication to the expansion of the *PmKIN* gene family could be ignored.

We identified 12 *PmKIN* genes located in discrete collinear segments through a collinearity analysis of the chromosomes. These findings revealed that segmental duplication was the main mechanism leading to the expansion of *PmKIN* family numbers. These duplication events, along with subsequent functional differentiation, have shaped the evolutionary diversity of the *PmKIN* family.

The analysis of Ka and Ks rates for six *PmKIN* homologous pairs enabled us to evaluate the evolutionary limitations and functional specialization trends within this family (Table 1). The Pm*KIN20*/*PmKIN41* pair exhibited the highest Ka (0.394) and Ks (1.72) values, while the *PmKIN28*/*PmKIN36* pair recorded the lowest values (Ka = 0.160, Ks = 1.19). Across all pairs examined, Ka/Ks ratios were less than one, demonstrating that the *PmKIN* family had purifying selection and strong functional conservation.

### 3.5. Interspecific Collinearity Analysis of the PmKIN Gene Family

**Figure 4:**
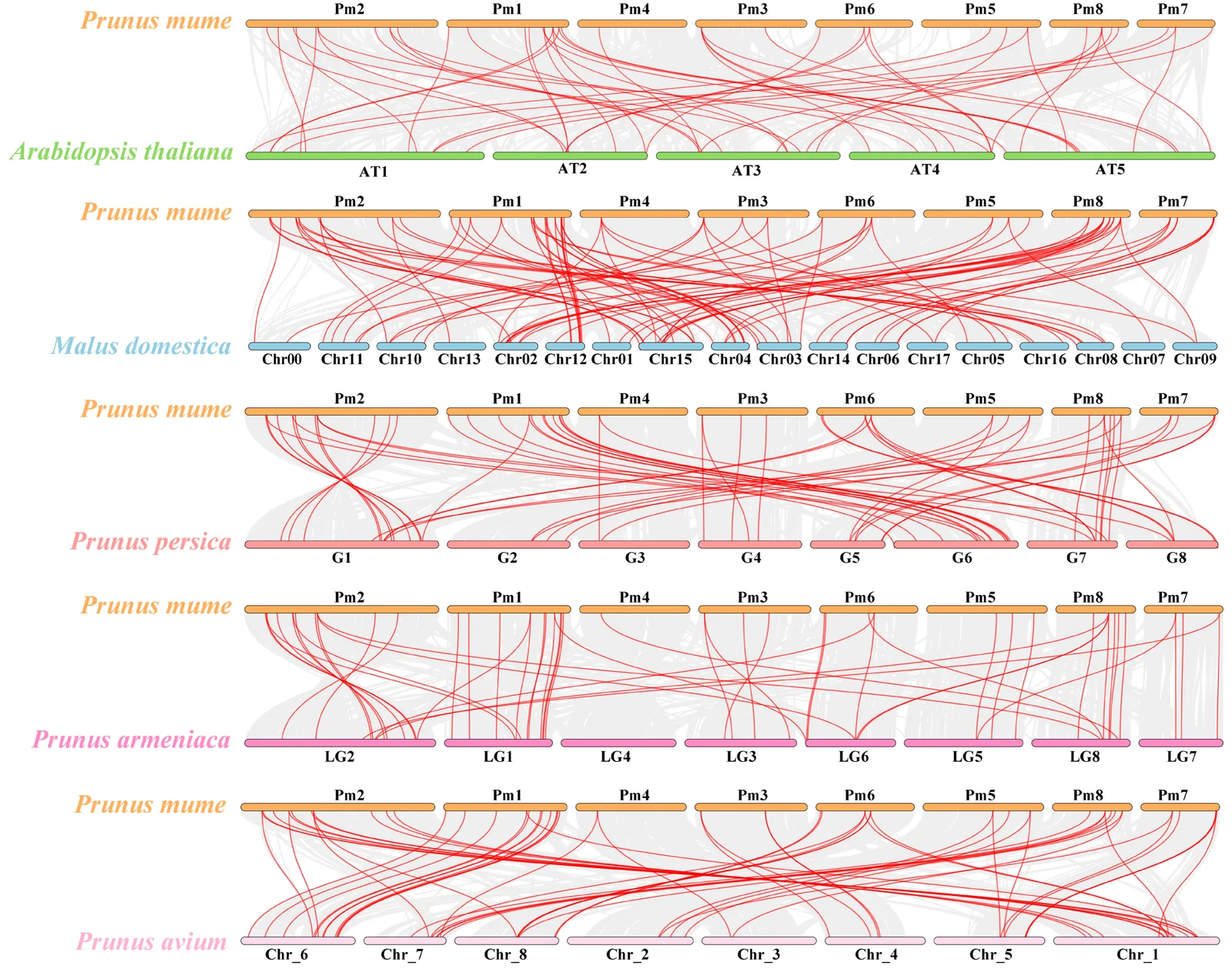
Interspecific synteny analysis of the *PmKIN* gene family across multiple plant species.

We employed interspecific collinearity analysis to systematically examine the evolutionary history and functional stability of the *PmKIN* gene family (Figure 4 and Supplementary Table S3). Collinearity alignment was carried out between the *PmKIN* gene family and their orthologs in *A. thaliana*, *P. persica*, *P. armeniaca*, *P. avium*, and *M. domestica*. The results identified 51, 65, 59, 67, and 99 homologous gene pairs between *P. mume* and *A. thaliana*, *P. persica*, *P. armeniaca*, *P. avium*, and *M. domestica*, respectively. These orthologs across different species share highly analogous biological functions. Interspecific collinearity results confirmed the high functional conservation of the *PmKIN* gene family during species evolution. Moreover, these findings further supported that gene duplication constituted the core driver for gene expansion and functional evolution of the *PmKIN* family in *P. mume*.

Comparative synteny was performed among *A. thaliana*, *M. domestica*, *P. persica*, *P. armeniaca*, and *P. avium*. Red connecting lines represent orthologous collinear gene pairs between *PmKIN* genes and their counterparts in other taxa.

### 3.6. Cis-Acting Element Analysis in the Promoters of the PmKIN Gene Family

**Figure 5:**
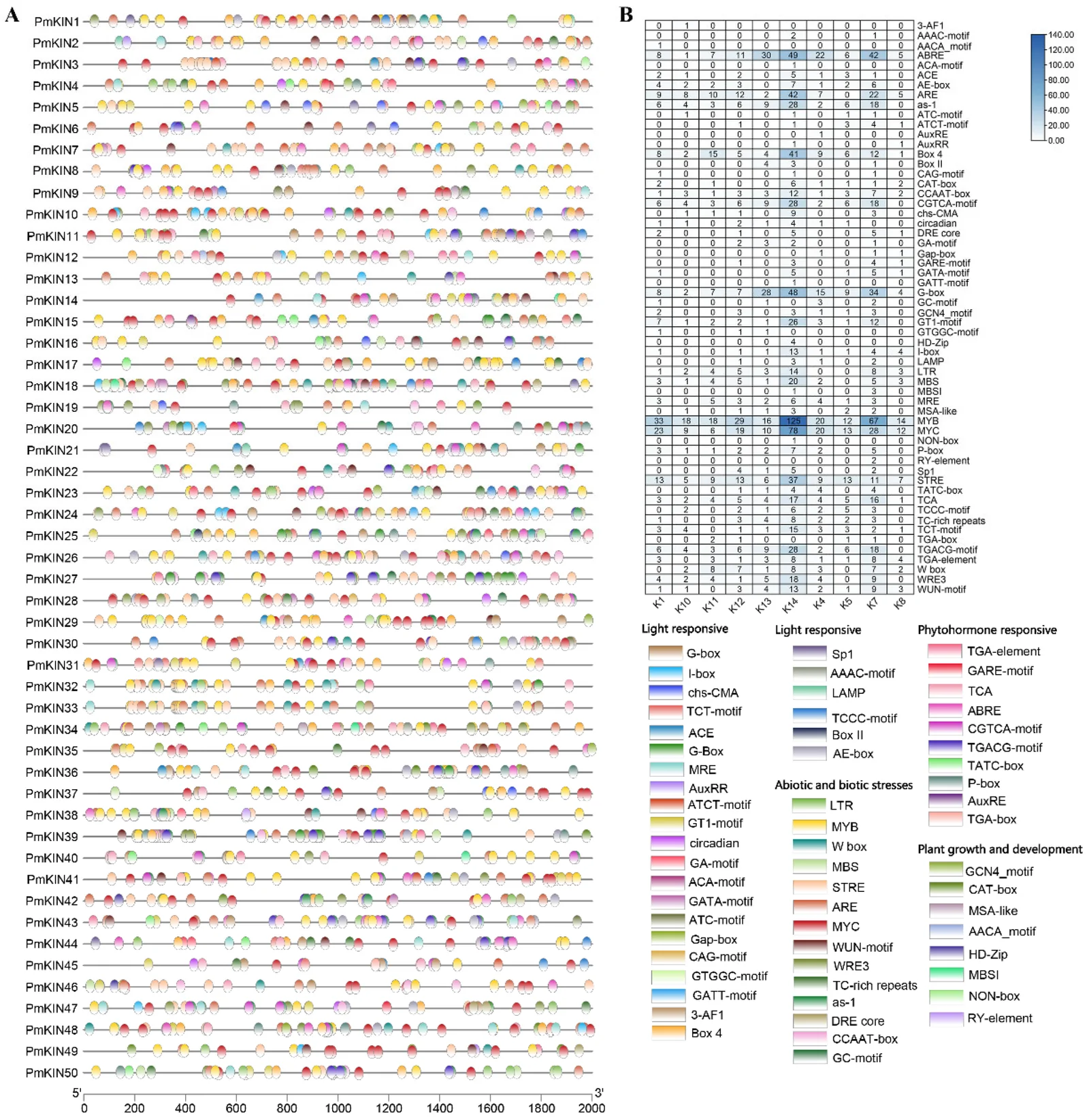
Predicted cis-regulatory elements within the promoter regions of *PmKIN* genes (**A**) and quantitative statistics of cis-element counts across distinct subfamilies (**B**).

Promoter sequences (2000 bp upstream of TSS) of all *PmKIN* genes were analyzed for cis-acting elements to uncover their potential regulatory functions (Figure 5A). The results revealed that the *PmKIN* promoters were enriched with four major classes of functional regulatory motifs, including elements responding to light, phytohormones, biotic and abiotic stresses, and plant growth and developmental signals. It was worth noting that cis-acting elements linked to light responses constituted the most diverse category, with various subtypes distributed in the promoter region of *PmKIN* members. Identified hormone-responsive motifs encompassed responsive sites for gibberellin (GARE-motif, TATC-box, and P-box), auxin (TGA-element, AuxRE, and TGA-box), abscisic acid (ABRE), salicylic acid (TCA), and jasmonic acid (CGTCA-motif and TGACG-motif). Development-associated cis-elements covered modules governing meristem expression, endosperm development, cell cycle control, vegetative growth, and seed-specific expression. Cis-elements for biotic and abiotic stresses covered low-temperature-responsive, drought-responsive, oxidative stress-responsive, and wound-responsive motifs, alongside conserved elements involved in defense and stress responses.

The composition of cis-elements showed significant differences in subfamily specificity, with K14 containing the most abundant promoter cis-elements (Figure 5B). Light-responsive motifs occurred most frequently in *PmKIN* promoters, with the K14 subfamily displaying higher enrichment than all other subfamilies. Broad distribution was observed for phytohormone-responsive elements. ABRE occupied most *PmKIN* promoters, and its copy number was significantly higher in K14 and K7 than in other subfamilies, whereas K13 and K4 also contained abundant ABRE. Several subfamilies (K14 and K8) were characterized by abundant CGTCA-motif. Cis-elements related to plant growth and development existed widely among diverse *PmKIN* subfamilies. Cis-elements responsive to abiotic stresses displayed broad distribution across *PmKIN* subfamilies and peaked within K14, which harbored 125 *MYB* and 78 *MYC* binding sites. The K7 subfamily also accumulated considerable quantities of these motifs. Collectively, these observations implied that K14 and K7 *PmKIN* members are modulated by *MYB*/*MYC* transcription factors and have an important function in cold, drought, and other abiotic stress signaling pathways. In addition, LTR cold-responsive elements occurred widely in diverse subfamilies. Compared with other clades, subfamilies K14, K4, and K7 harbored abundant LTR cis-motifs.

### 3.7. Tissue Expression Patterns of the PmKIN Gene Family

**Figure 6:**
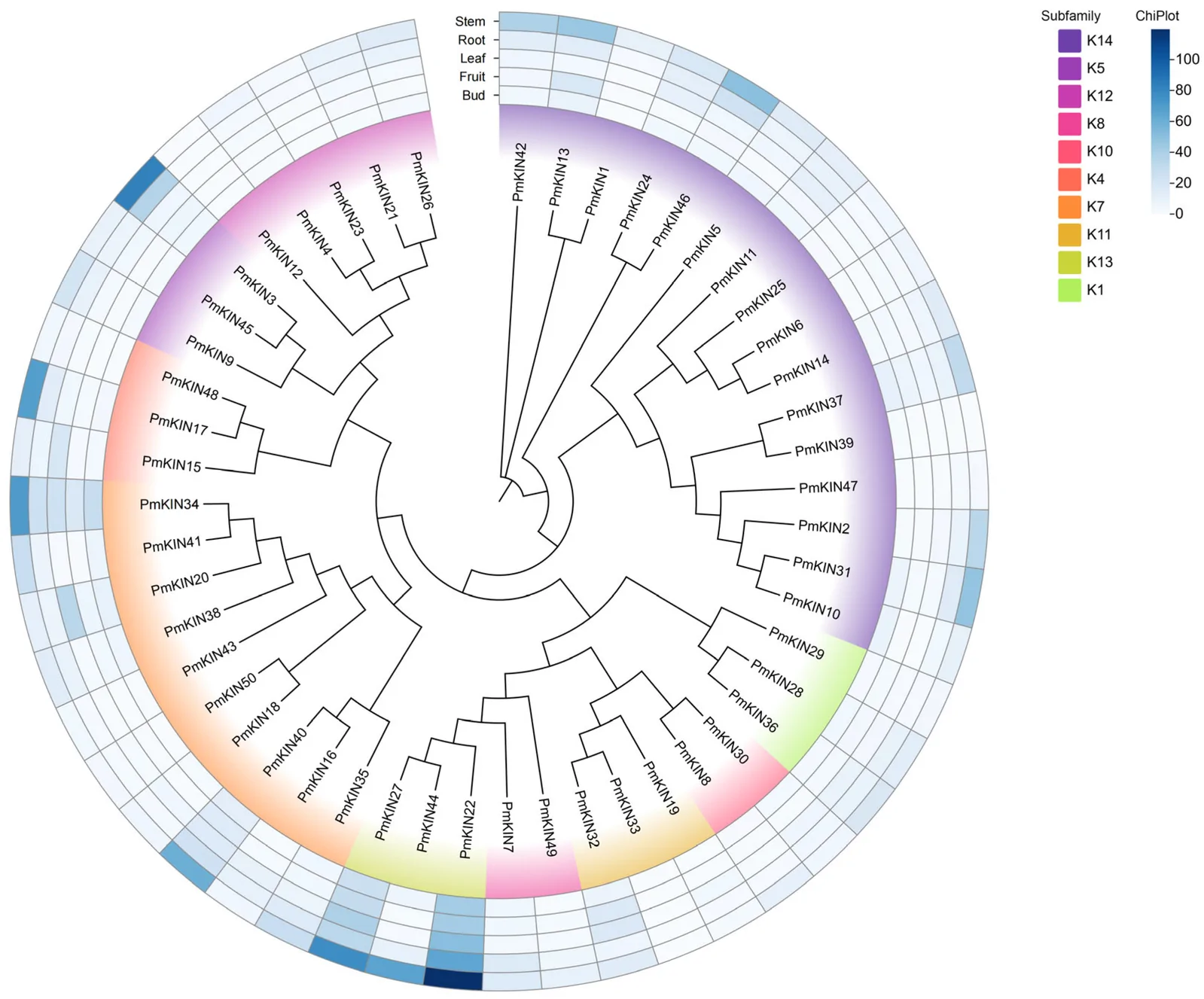
Expression patterns of *PmKIN* across various tissues.

To unravel the spatial expression features of *PmKIN* family members, their transcriptional abundances were surveyed in five organs, including the root, stem, leaf, fruit, and bud. The heatmap (Figure 6 and Supplementary Table S4) revealed that *PmKIN* genes exhibited prominent subfamily-specific expression and obvious tissue expression divergence. The highest transcriptional accumulation of the *PmKIN* family occurred in roots and stems. *PmKIN22* and *PmKIN27* exhibited high transcript levels in stems, roots, leaves, and buds. By contrast, most *PmKIN* genes were expressed at low levels in fruits. Collectively, distinct expression patterns were detected for individual *PmKIN* genes across the examined tissues, suggesting that members of this family may exert divergent functions during the development of roots, stems, leaves, fruits, and buds.

The outer panel depicts the expression heatmap of *PmKIN* genes across five tissues, namely the stem, root, leaf, fruit, and bud, ordered vertically. The inner cladogram corresponds to the phylogenetic tree of the *PmKIN* gene family, with individual subfamilies highlighted in different colors.

### 3.8. Expression Profile of the PmKIN Gene Family

**Figure 7:**
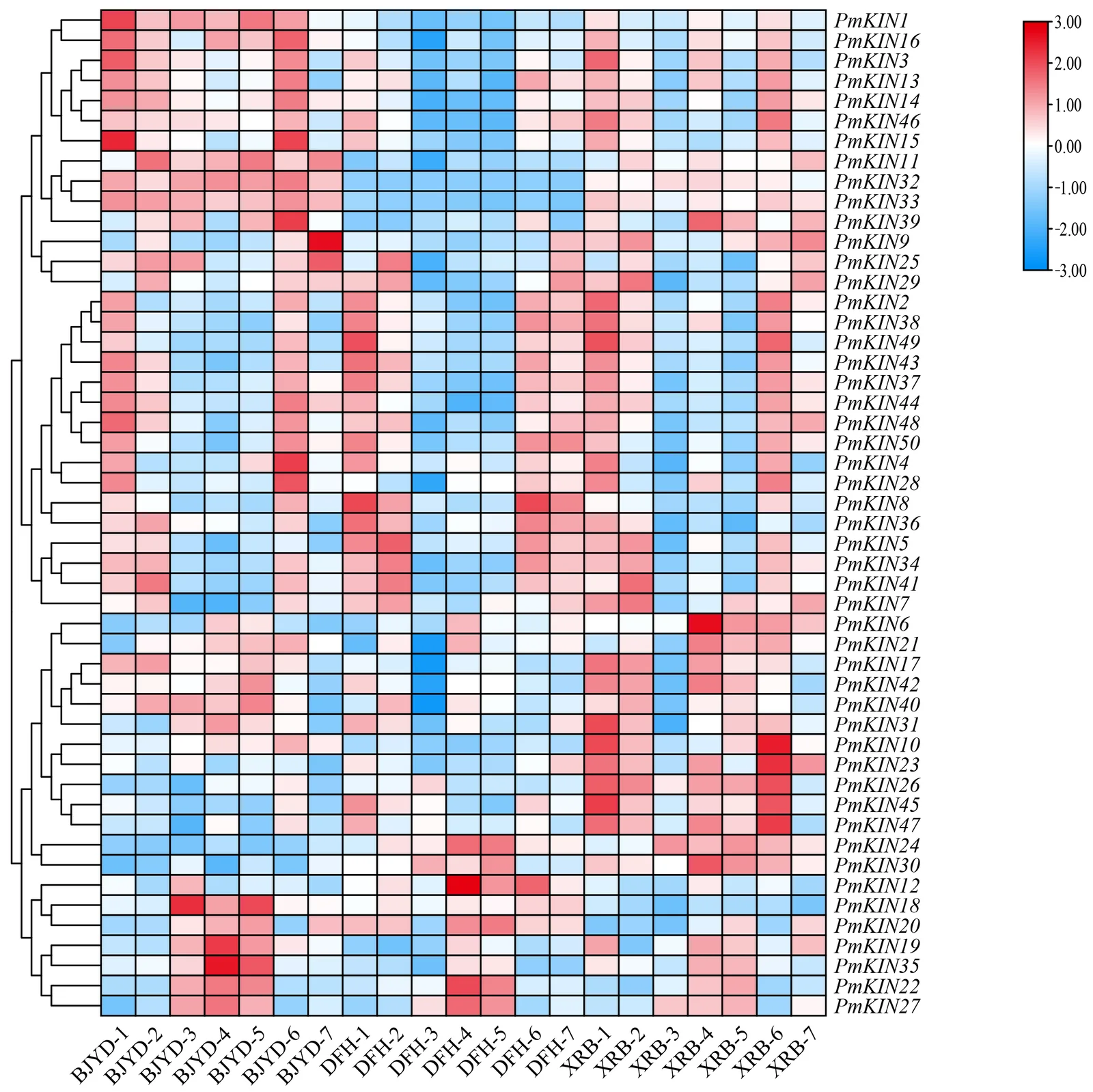
Hierarchical clustering analysis of *PmKIN* expression patterns under low-temperature stress across ‘BJYD’, ‘DFH’, and ‘XRB’.

We analyzed the expression patterns of the *PmKIN* gene family in three *P. mume* cultivars, namely ‘DFH’, ‘BJYD’, and ‘XRB’, following different low-temperature stress treatments and temperature recovery processes. Results revealed that the transcript levels of *PmKIN* genes differed significantly among temperatures, treatment durations, and tested genotypes (Figure 7 and Supplementary Table S5). After short-term low-temperature treatment at 4 °C for 8 h, most *PmKIN* genes were rapidly upregulated, among which *PmKIN13*, *PmKIN14*, and *PmKIN22* displayed remarkable induction exclusively in ‘DFH’ and ‘XRB’. These two genotypes outperformed ‘BJYD’ in both the number and expression magnitude of induced genes. This demonstrated that rapid significant activation of *PmKIN* was associated with prompt low-temperature responses in tolerant lines. Following 5 days of continuous 4 °C cold exposure, fewer *PmKIN* genes remained upregulated compared with short-term treatment. Most *PmKIN* genes (e.g., *PmKIN1* and *PmKIN3*) recovered to control levels post-treatment. ‘DFH’ and ‘XRB’ maintained high expression of multiple *PmKINs*, whereas ‘BJYD’ returned to basal levels. These expression differences are consistent with the previously reported cold-tolerance variation among the three genotypes.

The transcriptional profiles of *PmKIN* genes became significantly polarized after 1 h exposure to −5 °C freezing conditions. Specifically, *PmKIN15* and *PmKIN35* were strongly induced and *PmKIN10* and *PmKIN28* were transcriptionally suppressed, with few genes maintaining stable expression. Compared with ‘BJYD’, ‘DFH’ and ‘XRB’ had more induced *PmKIN* transcripts, which suggested that these genes may be involved in genotype-dependent cold response. During the recovery at 4 °C following freezing stress, the expression of most genes (*PmKIN2* and *PmKIN5*) returned to baseline levels under normal temperature control. In contrast, only a few members (*PmKIN11* and *PmKIN49*) maintained altered expression, indicating genotype-specific recovery dynamics. Transcription recovered much more completely in ‘DFH’ and ‘XRB’ close to basal expression, whereas partial genes from ‘BJYD’ persisted in dysregulated transcription. These discrepancies suggest that cold-resistant lines exhibit a more rapid restoration of transcript abundance toward basal levels during recovery, possibly reflecting a stronger recovery response to low-temperature stress. *PmKIN* members are responsive to diverse cold intensities, treatment durations, and recovery conditions. *PmKIN22*, *PmKIN27*, and *PmKIN35* may serve as candidate genes associated with low-temperature-responsive regulation, providing resources for further cold-tolerance studies.

B stood for ‘BJYD’, D for ‘DFH’, and X for ‘XRB’. The numbers 1–7 represent the following treatments: (1) stems treated at 25 °C; (2) stems subjected to 4 °C low-temperature treatment for 8 h; (3) stems treated at 4 °C for 5 days; (4) stems exposed to −5 °C freezing stress for 1 h; (5) samples recovered from −5 °C back to 4 °C; (6) non-acclimated stems treated at −5 °C for 1 h; and (7) non-acclimated stems transferred from −5 °C to 4 °C for recovery.

### 3.9. Expression Analysis of the PmKIN Gene Family in Response to Low-Temperature Stress

**Figure 8:**
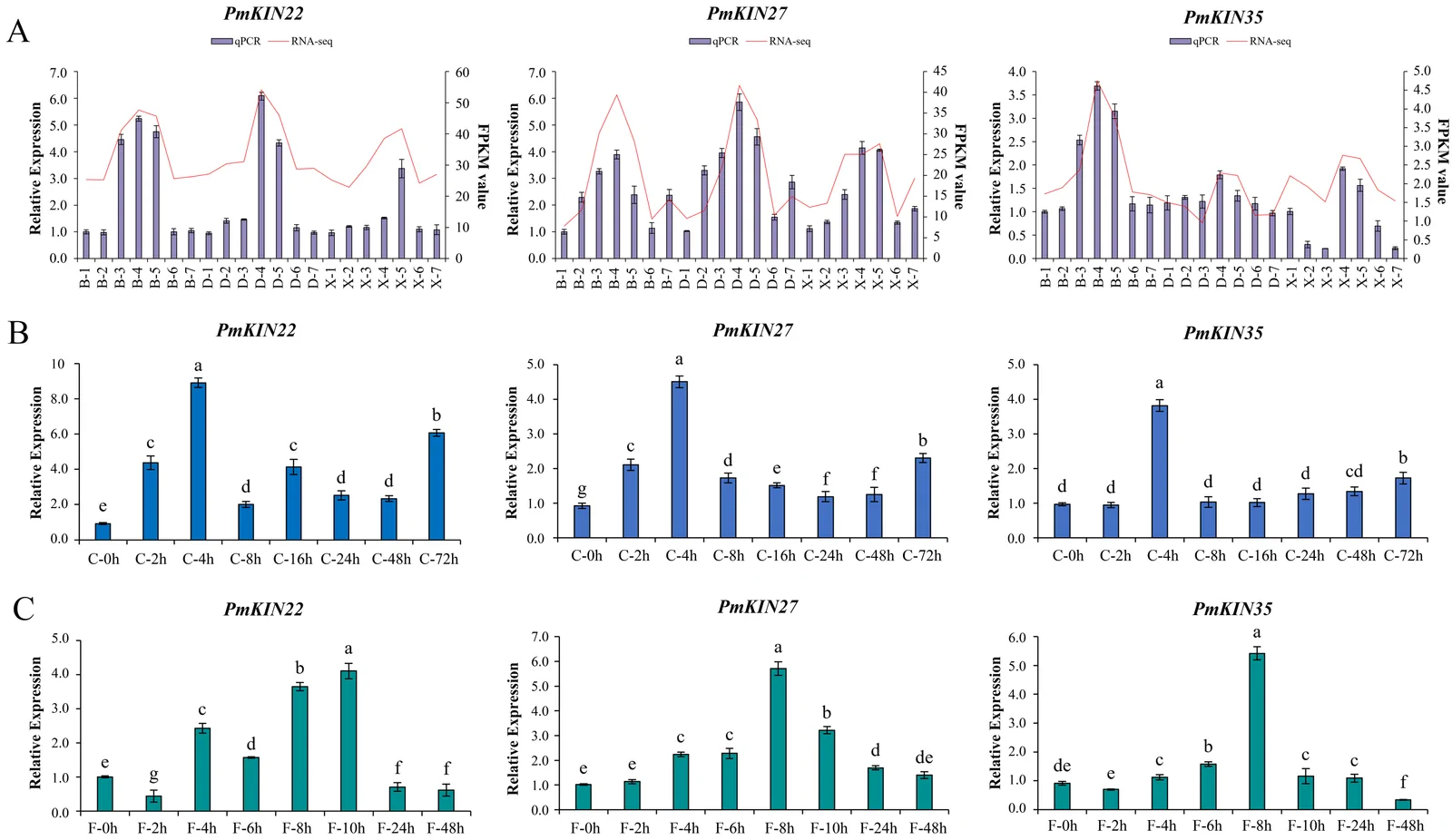
Analysis of the expression levels of *PmKINs* under low-temperature stress. (**A**) Expression levels of PmKIN genes in cultivars ‘BJYD’, ‘DFH’, and ‘XRB’ under low-temperature stress. (**B**) Transcriptional dynamics of *PmKINs* upon cold exposure in ‘BJYD’. (**C**) Transcriptional dynamics of *PmKINs* upon freezing exposure in ‘BJYD’. ‘C’ and ‘F’ are abbreviations for cold and freezing treatment, respectively. Dissimilar letters denote significant differences among groups (*p* < 0.05). Error bars represent standard deviation (SD).

In this study, qRT-PCR was applied to systematically measure the relative expression levels of three key genes, *PmKIN22*, *PmKIN27*, and *PmKIN35*, under low-temperature stress with different time-series treatments. Their transcriptional expression patterns responding to low-temperature stress were analyzed. The time gradients for cold stress treatment were set to 0, 2, 4, 8, 16, 24, 48, and 72 h, while those for freezing stress treatment were 0, 2, 4, 6, 8, 10, 24, and 48 h. Consistent with the transcriptome data, the expression profiles of *PmKIN22*, *PmKIN27*, and *PmKIN35* were validated via qRT-PCR analysis under low-temperature conditions (Figure 8A).

Under cold-stress exposure, the transcript levels of the three genes were maximally induced at the 4 h time point (Figure 8B). Among them, *PmKIN22* was generally upregulated upon induction, followed by fluctuating expression, with significantly elevated expression again at the late stage of stress treatment. *PmKIN27* peaked in transcription at 4 h, then its expression gradually decreased, with a rebound observed at 72 h. *PmKIN35* exhibited specific and significant upregulation solely at 4 h. Its transcript levels showed no significant differences compared with the initial control group at all other time points, with only a slight elevation detected at 72 h. Following freezing stress treatment, *PmKIN22* was transiently downregulated at the early stage of stress. Then its expression was continuously upregulated and peaked at F-10 h, followed by a sharp reduction at F-24 h and F-48 h (Figure 8C). The transcript accumulation of *PmKIN27* rose initially and declined thereafter as freezing stress proceeded, with peak expression detected at 8 h, and its transcript abundance decreased progressively at subsequent time points. Transcriptional induction of *PmKIN35* was specifically restricted to the 8 h time point during freezing treatment, where its transcript level spiked drastically. Mild fluctuations were observed throughout the remaining time series, and its expression declined to the minimum value of the entire treatment group by 48 h. Collectively, these findings demonstrate that *PmKIN22*, *PmKIN27*, and *PmKIN35* are transcriptionally inducible upon cold and freezing stimuli in *P. mume*.

### 3.10. Protein–Protein Interaction Network Analysis

**Figure 9:**
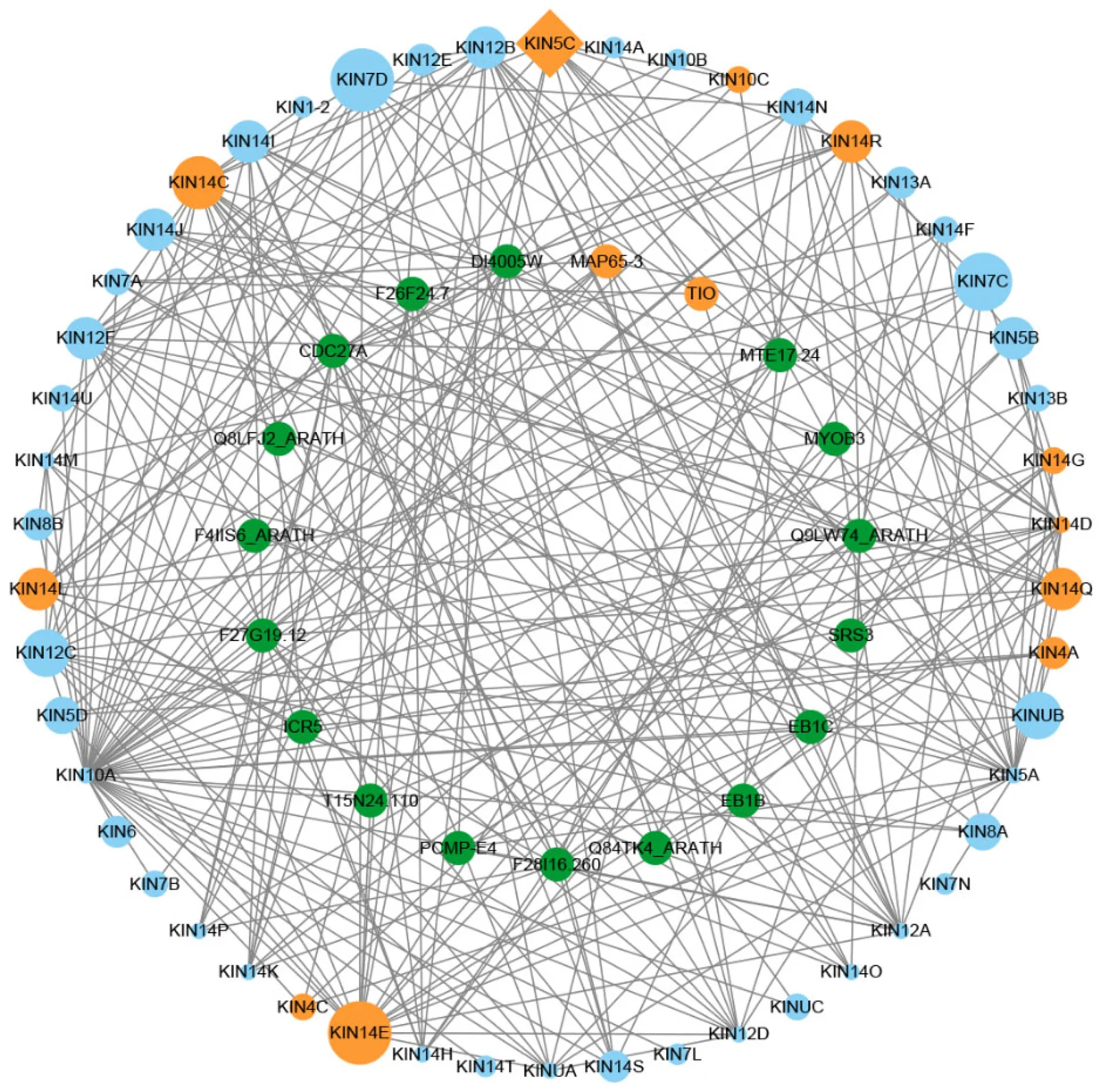
Interaction network of PmKIN proteins. Blue nodes correspond to *Arabidopsis* orthologs of PmKIN members and additional interacting proteins from *A. thaliana*. Yellow nodes mark core PmKIN proteins together with their interacting counterparts. Green nodes represent other predicted interacting protein partners derived from *A. thaliana*.

To gain insight into the functional partnerships and regulatory circuitry of *PmKIN* proteins, we generated a protein–protein interaction network using *Arabidopsis* kinesin homologs as the reference (Figure 9). The *A. thaliana* genome yielded orthologous counterparts for 43 PmKIN members, from which we predicted a total of 285 pairwise interactions. Interrogation of this network revealed KIN5C (PmKIN45) as the predominant hub, which interacts closely with 10 PmKIN members (KIN4A, KIN10C, KIN14C, KIN14D, etc.) and occupies a highly central position. The dense interaction patterns might point to synergistic regulation among kinesin proteins. They may coordinate core biological pathways through assembly into homodimers and multimeric complexes [31,32].

The network further linked PmKIN proteins to multiple AtPIN members, as well as MYOB3 and SRS3. Such cross-species interaction conservation may suggest the evolutionary stability of kinesin-mediated regulatory networks in angiosperms. Further analyses identified multiple significantly enriched proteins potentially associated with cytoskeleton regulation and signal transduction in the PmKIN interaction network, such as MAP65-3, MTE17.24, and T15N24.110. Other enriched candidates that may participate in microtubule dynamics and cell cycle progression included EB1B, EB1C, ICR5, and TIO [33,34]. It is speculated that PmKIN family proteins may integrate multiple intracellular signaling pathways to mediate plant responses to abiotic stresses, including drought, salinity, and low temperature, and may modulate endogenous auxin and gibberellin. Overall, the PmKIN interaction network may provide a valuable theoretical basis and research clues for further exploring the molecular functions of the kinesin gene family and elucidating its potential regulatory mechanisms underlying stress responses in *P. mume*.

## 4. Discussion

### 4.1. Evolutionary Features and Segmental Duplication Driving Expansion of the PmKIN Family

Kinesins are indispensable molecular motor proteins prevalent across the plant kingdom, which power energy transduction via ATP hydrolysis to orchestrate an array of fundamental cellular activities ranging from intracellular cargo trafficking and cytoskeletal dynamic remodeling to mitotic progression and sophisticated signal transduction cascades [35,36]. Accumulating evidence in recent years has demonstrated that this gene family functions as a central regulator in response to abiotic stresses, particularly low-temperature stress [13,16,37]. Genome-wide surveys have systematically identified members of the kinesin gene family across diverse plant species, with 61 in *A. thaliana* [31], 139 in *Glycine max* [18], 48 in *Citrullus lanatus* [38], 155 in *T. aestivum* [20], and 172 in *Gossypium hirsutum* [15]. Nevertheless, systematic investigations of the phylogenetic evolution, molecular characteristics, and stress tolerance functions of the *PmKIN* gene family remain largely insufficient to date.

Genome-wide identification revealed 50 *PmKIN* members in *P. mume*, which were phylogenetically grouped into ten distinct subfamilies (K1, K4, K5, K7, K8, K10, K11, K12, K13, and K14). *PmKIN* subfamily classification aligns well with that of *A. thaliana*, confirming that kinesin genes are structurally and evolutionarily conserved across land plants [31]. All PmKIN proteins possess the conserved kinesin motor domain, a characteristic structural unit widespread throughout the kinesin superfamily. The preserved core domain might enable ATP-powered motility along microtubules in *P. mume*. Additionally, variable C-terminal sequences might endow *PmKIN* genes with different functions like in *Arabidopsis* [31], but *PmKINs* lack relevant experimental evidence.

The chromosomal distribution of *PmKIN* genes was uneven. In total, 13 genes gathered on chromosome Pm1, while other chromosomes only contained scattered genes. We observed abundant segmental duplication without tandem repeat via collinearity screening. This demonstrated segmental genome duplication primarily drove the proliferation of *PmKIN* family members. Similarly, studies on *C. lanatus* [38] and *G. max* [18] have shown that segmental duplication promotes kinesin gene amplification in a conserved manner. Concentrated *PmKIN* copies on Pm1 may come from ancient duplicated chromosomal blocks, but the functional significance of this gene aggregation is uncharacterized. In the future, researchers can focus on whether these clustered paralogs work synergistically or evolve divided biological functions. We further performed interspecific synteny analysis and found many homologous regions shared between the genome of *P. mume* and other Rosaceae species. The abundant homologous sites and highly conserved synteny suggest that the kinesin family maintains stable structural and functional integrity during the differentiation process of Rosaceae species.

The *PmKIN* family was mainly subjected to purifying selection (Ka/Ks < 1) during its evolutionary process. Strong purifying selection pressure was also prevalent during the evolutionary progression of *KIN* genes in *G. hirsutum*, *Pisum sativum*, and other plant species [15,37]. However, the strength of selective constraints differed across different copy pairs within the *PmKIN* family. The heterogeneity of selection pressure indicates that different homologous copies experience varying degrees of evolutionary constraints after replication.

### 4.2. Cis-Regulatory Elements and Tissue Expression Reveal Signal Response and Functional Divergence in PmKIN Family

Promoter cis-acting elements function as core regulatory modules that control gene spatiotemporal expression and environmental signal perception in plants. Notably, *PmKIN* promoters were widely enriched with functional cis-motifs that mediated light signaling, phytohormone response, plant development, and biotic/abiotic stress tolerance. In detail, cold-responsive LTR elements and stress-related MYB and MYC binding sites were significantly enriched in most cold-tolerant *PmKIN* candidates, providing insights into the transcriptional molecular basis of *PmKIN*-mediated low-temperature-stress responses in *P. mume*. This regulatory feature mirrors the enrichment of stress-responsive elements in the promoter of the rice cold-tolerant kinesin *OsDLK* [16], indicating a conserved transcriptional activation mechanism of kinesin families underlying plant low-temperature-stress responses.

Tissue-specific expression patterns provide intuitive evidence for functional divergence within gene families. Several *PmKIN* genes showed dominant transcription in root and stem tissues, likely responsible for cytoskeleton homeostasis and intracellular cargo transport. Such biological roles were in accordance with documented evidence that *AtKINESIN-13A* drove microtubule dynamic rearrangement within vegetative organs [39]. Other *PmKIN* genes exhibited high-abundance expression in flower buds, implying their regulatory roles in reproductive-related processes. Distinct from the leaf-specific cold responsiveness of rice *OsDLK*, the preferential expression of *PmKIN* in floral organs reveals special adaptive demands of the reproductive structures to freezing stress in woody perennials [16]. Most *PmKIN* genes exhibited relatively low transcriptional levels in fruit tissues. This expression profile is well matched to the species-specific biological feature of *P. mume*, which was mainly cultivated for its ornamental flowers. The above tissue-specific expression characteristics are similar to the tissue-preferential expression pattern of the *PsKIN* gene family in pea under drought stress [37], which further confirms that the functional differentiation of kinesin families in different plant tissues is evolutionarily conserved across species.

### 4.3. Temporal Expression of PmKIN Modulates Low-Temperature Responses in P. mume

Cold stress constrains plant biogeographical distribution, suppresses photosynthesis and metabolic processes, and ultimately inhibits plant growth, development, reproductive performance, and yield formation [40]. Our results revealed that the cold-resistant genotypes ‘DFH’ and ‘XRB’ could trigger rapid transcriptional activation of a larger set of *PmKIN* genes upon exposure to short-duration cold stress (4 °C, 8 h) and severe freezing treatment (−5 °C, 1 h). The fold changes in gene upregulation were markedly greater in these two tolerant lines relative to the susceptible ‘BJYD’ line, accompanied by a superior ability to revert transcript abundance back to the basal level during the recovery phase. *PmKIN* genes in cold-resistant lines displayed a characteristic expression profile featuring rapid induction, robust transcriptional expression, and fast recovery.

Meanwhile, we performed quantitative verification via time-series gradient qRT-PCR on the core responsive genes *PmKIN22*, *PmKIN27*, and *PmKIN35* screened in this study. The three genes were markedly induced under both cold and freezing stress. Notably, their temporal response patterns, peak expression stages, and dynamic fluctuation trends differed significantly, revealing obvious gene-specific characteristics and stress-type specificity. This study offers a new mechanistic insight into the low-temperature responses of woody plants from the perspective of cytoskeleton dynamics. Conventional cold-induced proteins (e.g., COR and LEA proteins) are generally regarded as a type of passive protector which function to stabilize membrane structures and protein conformations via non-enzymatic and energy-independent pathways [41,42]. As ATP-dependent active molecular motors, kinesins undergo rapid transcriptional activation, implying that plants rely not only on passive defense under cold stress but also actively trigger cytoskeleton rearrangement and the reprogramming of material transport pathways. This finding aligns with the emerging trend that kinesins have evolved functional roles from cargo transporters to signal regulators [16]. These findings further verify that *PmKIN* genes in cold-resistant lines present a programmed expression characteristic, including rapid stress induction, temporally segmented high-efficiency expression, and prompt expression restoration upon stress removal in *P. mume*.

### 4.4. Interaction Network of PmKIN Genes Regulates Microtubule Cytoskeleton and Stress Signaling Pathways

In this study, we firstly systematically constructed the protein–protein interaction network of the *PmKIN* family in *P. mume*, which clarified the potential intracellular topological interaction structure of these family proteins at the systematic level. A total of 43 PmKIN proteins were assigned to *Arabidopsis* orthologs, yielding 285 interacting pairs, which highlights remarkable genomic integrity and evolutionary functional conservation of the *PmKIN* family [31]. PmKIN45 (KIN5C) was identified as a core hub in the protein–protein interaction network, which can form a densely interconnected interaction cluster with ten gene family members. Considering the well-documented functions of its homologous proteins in spindle assembly and chromosome movement in *A. thaliana* [43], we speculate that PmKIN45 might act as a core organizer coordinating motor protein activity during cell division in *P. mume*. Furthermore, PmKIN proteins might execute biological functions via assembling homodimeric, heterodimeric, and multimeric complexes.

Current interaction data suggest that PmKIN proteins may physically interact with MYOB3 and SRS3. These putative interactions potentially provide a preliminary molecular clue for the probable roles of the *PmKIN* family in actin cytoskeleton homeostasis, mechanical stress perception, and developmental signal transduction. The *PmKIN* interaction network is putatively enriched in key regulatory factors, including MAP65-3, EB1B/C, ICR5, and TIO. In particular, functional studies on *Arabidopsis* have demonstrated that MAP65-3 selectively cross-links antiparallel microtubules in the phragmoplast. This microtubule-binding protein is required for the recruitment of Kinesin-12 and other motor proteins to microtubule plus ends [44]. ICR5 functions as a microtubule-associated effector downstream of the ROP (Rho of Plants) pathway, modulating microtubule dynamics and secondary cell wall deposition [45]. Collectively, the PmKIN interaction network implies that PmKIN proteins may potentially coordinate microtubule bundle homeostasis, dynamic remodeling, and physical stimulus perception. Meanwhile, PmKIN proteins might bridge the ROP signaling cascade and secretory pathway, thereby potentially integrating auxin and gibberellin hormonal signaling.

## 5. Conclusions

This study identified 50 *PmKIN* members in *P. mume* and categorized them into 10 subfamilies. We confirmed that segmental duplication served as the major contributor to the expansion of the *PmKIN* family. Additionally, this family underwent purifying selection pressure and was evolutionarily conserved within Rosaceae plants. Divergences were observed in the gene structure and promoter cis-acting elements of this gene family. A large number of cold-responsive regulatory elements were enriched in these sequences, and the genes displayed organ-specific expression patterns across different tissues. Low-temperature stress assays imply that *PmKIN22*, *PmKIN27*, and *PmKIN35* may serve as candidate genes associated with low-temperature-response regulation. PmKIN45 might act as the central hub within the protein interactome, potentially coordinating the modulation of cytoskeleton dynamics, vegetative development, and cold-responsive signal transduction pathways. These findings provide candidate genes for cold-resistance molecular breeding and lay a theoretical foundation for expanding the northern cultivation range of *P. mume*.

## Figures and Tables

**Table 1:** Selection pressure (Ka/Ks) analysis of *PmKIN* paralogous gene pairs.

Gene Pairs	Gene Pairs	Ka	Ks	Ka/Ks	Divergence Time (Mya)
PmKIN6	PmKIN14	0.289	1.43	0.20	47.64
PmKIN20	PmKIN34	0.373	1.62	0.23	53.93
PmKIN20	PmKIN41	0.394	1.72	0.23	57.44
PmKIN27	PmKIN44	0.176	1.27	0.14	42.20
PmKIN28	PmKIN36	0.160	1.19	0.13	39.60
PmKIN34	PmKIN41	0.278	1.46	0.19	48.56

## Data Availability

The original contributions presented in this study are included in the article and Supplementary Materials. Further inquiries can be directed to the corresponding author.

## Supplementary Materials

The following supporting information can be downloaded at: https://www.mdpi.com/article/10.3390/genes17091151/s1. Supplementary Figure S1. Chromosomal localization of the *PmKIN* gene family. Supplementary Table S1. Primer sequences used for quantitative real-time PCR analysis. Supplementary Table S2. Feature profiles of all characterized *PmKIN* genes in *P. mume*. Supplementary Table S3. Genomic collinearity fragments corresponding to *PmKIN* genes. Supplementary Table S4. Tissue FPKM profiles of *PmKIN* genes in wild *P. mume*. Supplementary Table S5. RPKM transcript levels of *PmKIN* genes subjected to low-temperature stress.

## References

[1] Ding Y., Shi Y., Yang S. (2024). Regulatory Networks Underlying Plant Responses and Adaptation to Cold Stress. Annu. Rev. Genet..

[2] Peng Y., Ming Y., Jiang B., Zhang X., Fu D., Lin Q., Zhang X., Wang Y., Shi Y., Gong Z. (2024). Differential phosphorylation of Ca^2+^-permeable channel CYCLIC NUCLEOTIDE-GATED CHANNEL20 modulates calcium-mediated freezing tolerance in Arabidopsis. Plant Cell.

[3] Dong B., Zheng Z., Zhong S., Ye Y., Wang Y., Yang L., Xiao Z., Fang Q., Zhao H. (2022). Integrated Transcriptome and Metabolome Analysis of Color Change and Low-Temperature Response during Flowering of *Prunus mume*. Int. J. Mol. Sci..

[4] Miao R., Li M., Wen Z., Meng J., Liu X., Fan D., Lv W., Cheng T., Zhang Q., Sun L. (2023). Whole-Genome Identification of Regulatory Function of *CDPK* Gene Families in Cold Stress Response for *Prunus mume* and *Prunus mume* var. Tortuosa. Plants.

[5] Zhao K., Zhou Y., Li Y., Zhuo X., Ahmad S., Han Y., Yong X., Zhang Q. (2018). Crosstalk of PmCBFs and PmDAMs Based on the Changes of Phytohormones under Seasonal Cold Stress in the Stem of *Prunus mume*. Int. J. Mol. Sci..

[6] Huang X., Gao F., Zhou P., Ma C., Tan W., Ma Y., Li M., Ni Z., Shi T., Hayat F. (2024). Allelic variation of PmCBF03 contributes to the altitude and temperature adaptability in Japanese apricot (*Prunus mume* Sieb. et Zucc.). Plant Cell Environ..

[7] Zhang Y., Dong G., Chen F., Xiong E., Liu H., Jiang Y., Xiong G., Ruan B., Qian Q., Zeng D. (2022). The kinesin-13 protein BR HYPERSENSITIVE 1 is a negative brassinosteroid signaling component regulating rice growth and development. Theor. Appl. Genet..

[8] Gicking A.M., Swentowsky K.W., Dawe R.K., Qiu W. (2018). Functional diversification of the kinesin-14 family in land plants. FEBS Lett..

[9] Nadeem M., Chen A., Hong H., Li D., Li J., Zhao D., Wang W., Wang X., Qiu L. (2021). GmMs1 encodes a kinesin-like protein essential for male fertility in soybean (*Glycine max* L.). J. Integr. Plant Biol..

[10] Chen G., Xuan W., Zhao P., Yao X., Peng C., Tian Y., Ye J., Wang B., He J., Chi W. (2022). OsTUB1 confers salt insensitivity by interacting with Kinesin13A to stabilize microtubules and ion transporters in rice. New Phytol..

[11] Li J., Yu D., Qanmber G., Lu L., Wang L., Zheng L., Liu Z., Wu H., Liu X., Chen Q. (2019). GhKLCR1, a kinesin light chain-related gene, induces drought-stress sensitivity in Arabidopsis. Sci. China Life Sci..

[12] Yang X.Y., Chen Z.W., Xu T., Qu Z., Pan X.D., Qin X.H., Ren D.T., Liu G.Q. (2011). Arabidopsis kinesin KP1 specifically interacts with VDAC3, a mitochondrial protein, and regulates respiration during seed germination at low temperature. Plant Cell.

[13] Xu X., Walter W.J., Liu Q., Machens I., Nick P. (2018). A rice class-XIV kinesin enters the nucleus in response to cold. Sci. Rep..

[14] Xing X., Liu M., Jiang F., Zhou R., Bai Y., Wei H., Zhang D., Wei J., Wu Z. (2022). Abscisic acid induces the expression of AsKIN during the recovery period of garlic cryopreservation. Plant Cell Rep..

[15] Gu L., Wei F., Chen P., Yang M., Liu Z. (2023). Genome-wide identification and characterization of the kinesin gene family in upland cotton (*Gossypium hirsutum* L.). Ind. Crops Prod..

[16] Xu X., Hummel S., Harter K., Kolukisaoglu U., Riemann M., Nick P. (2022). The Minus-End-Directed Kinesin OsDLK Shuttles to the Nucleus and Modulates the Expression of Cold-Box Factor 4. Int. J. Mol. Sci..

[17] Zhang Q., Chen W., Sun L., Zhao F., Huang B., Yang W., Tao Y., Wang J., Yuan Z., Fan G. (2012). The genome of *Prunus mume*. Nat. Commun..

[18] Jin T., Zhang K., Zhang X., Wu C., Long W. (2025). Genome-Wide Identification of the Kinesin Gene Family in Soybean and Its Response to Salt Stress. Agronomy.

[19] Ding A., Bao F., Cheng W., Cheng T., Zhang Q. (2023). Phylogeny of *PmCCD* Gene Family and Expression Analysis of Flower Coloration and Stress Response in *Prunus mume*. Int. J. Mol. Sci..

[20] Chen Q., Ren Y., Yan Q., Zheng Z., Zhang G., Ma L., Song Q., Niu N. (2024). Genome-wide identification and expression analysis of the kinesin gene superfamily suggests roles in response to abiotic stress and fertility of wheat (*Triticum aestivum* L.). BMC Genom..

[21] Chen C., Wu Y., Li J., Wang X., Zeng Z., Xu J., Liu Y., Feng J., Chen H., He Y. (2023). TBtools-II: A “one for all, all for one” bioinformatics platform for biological big-data mining. Mol. Plant.

[22] Yang Q., Yuan C., Cong T., Wang J., Zhang Q. (2022). Genome-wide identification of three-amino-acid-loop-extension gene family and their expression profile under hormone and abiotic stress treatments during stem development of *Prunus mume*. Front. Plant Sci..

[23] Li Y., Huang Z., Jiang Z., Sun L., Li S. (2026). Systematic Characterization of the Monosaccharide Transporter (MST) Gene Family in Citrus and Identification of Candidate Members Associated with Sugar Accumulation. Horticulturae.

[24] Lescot M., Dehais P., Thijs G., Marchal K., Moreau Y., Van de Peer Y., Rouze P., Rombauts S. (2002). PlantCARE, a database of plant cis-acting regulatory elements and a portal to tools for in silico analysis of promoter sequences. Nucleic Acids Res..

[25] Shannon P., Markiel A., Ozier O., Baliga N.S., Wang J.T., Ramage D., Amin N., Schwikowski B., Ideker T. (2003). Cytoscape: A software environment for integrated models of biomolecular interaction networks. Genome Res..

[26] Chen C., Chen H., Zhang Y., Thomas H.R., Frank M.H., He Y., Xia R. (2020). TBtools: An Integrative Toolkit Developed for Interactive Analyses of Big Biological Data. Mol. Plant.

[27] Letunic I., Bork P. (2021). Interactive Tree Of Life (iTOL) v5: An online tool for phylogenetic tree display and annotation. Nucleic Acids Res..

[28] Ding A., Bao F., Ding A., Zhang Q. (2022). Cold hardiness of *Prunus mume* ‘Xiang Ruibai’ and its parents based on biological indexes and physical parameters. Forests.

[29] Ding A., Ma Z., Zhang T., Zheng T., Wang J., Cheng T., Bao F., Ding A., Zhang Q. (2026). Transcriptomic analysis reveals the differences in cold tolerance among mei varieties and overexpression of PmWRKY18 enhances cold tolerance in tobacco. Ind. Crops Prod..

[30] Wang T., Lu J., Xu Z., Yang W., Wang J., Cheng T., Zhang Q. (2014). Selection of suitable reference genes for miRNA expression normalization by qRT-PCR during flower development and different genotypes of *Prunus mume*. Sci. Hortic..

[31] Reddy A.S., Day I.S. (2001). Kinesins in the Arabidopsis genome: A comparative analysis among eukaryotes. BMC Genom..

[32] Vale R.D. (2003). The molecular motor toolbox for intracellular transport. Cell.

[33] Nebenfuhr A., Dixit R. (2018). Kinesins and Myosins: Molecular Motors that Coordinate Cellular Functions in Plants. Annu. Rev. Plant Biol..

[34] Bisgrove S.R., Lee Y.R., Liu B., Peters N.T., Kropf D.L. (2008). The microtubule plus-end binding protein EB1 functions in root responses to touch and gravity signals in Arabidopsis. Plant Cell.

[35] Shen Z., Collatos A.R., Bibeau J.P., Furt F., Vidali L. (2012). Phylogenetic analysis of the Kinesin superfamily from physcomitrella. Front. Plant Sci..

[36] Galindo-Trigo S., Grand T.M., Voigt C.A., Smith L.M. (2020). A malectin domain kinesin functions in pollen and seed development in Arabidopsis. J. Exp. Bot..

[37] Yuan H., Liu B., Zhang G., Feng Z., Wang B., Bu Y., Xu Y., Gong Y., Sun Z., Liu N. (2024). Genome-wide identification and expression analysis of the *PsKIN* gene family in pea. Front. Genet..

[38] Tian S., Jiang J., Xu G.Q., Wang T., Liu Q., Chen X., Liu M., Yuan L. (2021). Genome wide analysis of kinesin gene family in Citrullus lanatus reveals an essential role in early fruit development. BMC Plant Biol..

[39] Fujikura U., Elsaesser L., Breuninger H., Sanchez-Rodriguez C., Ivakov A., Laux T., Findlay K., Persson S., Lenhard M. (2014). Atkinesin-13A modulates cell-wall synthesis and cell expansion in Arabidopsis thaliana via the THESEUS1 pathway. PLoS Genet..

[40] Huang L.Q., Yang C., Iqbal A., Liu J.T., Li Y.K., Bao H.N., Liu H.J., Chen Y.L., Li J., Zhang K. (2025). Jasmonate pathway regulates sphingolipid desaturation during cold stress. New Phytol..

[41] Artus N.N., Uemura M., Steponkus P.L., Gilmour S.J., Lin C., Thomashow M.F. (1996). Constitutive expression of the cold-regulated Arabidopsis thaliana *COR15a* gene affects both chloroplast and protoplast freezing tolerance. Proc. Natl. Acad. Sci. USA.

[42] Gusain S., Joshi S., Joshi R. (2023). Sensing, signalling, and regulatory mechanism of cold-stress tolerance in plants. Plant Physiol. Biochem..

[43] Hirokawa N., Noda Y., Tanaka Y., Niwa S. (2009). Kinesin superfamily motor proteins and intracellular transport. Nat. Rev. Mol. Cell Biol..

[44] Ho C.M., Hotta T., Guo F., Roberson R.W., Lee Y.R., Liu B. (2011). Interaction of antiparallel microtubules in the phragmoplast is mediated by the microtubule-associated protein MAP65-3 in Arabidopsis. Plant Cell.

[45] Feiguelman G., Cui X., Sternberg H., Hur E., Higa T., Oda Y., Fu Y., Yalovsky S. (2022). Microtubule-associated ROP interactors affect microtubule dynamics and modulate cell wall patterning and root hair growth. Development.

